# Characterization of the urinary metabolic profile of cholangiocarcinoma in a United Kingdom population

**DOI:** 10.2147/HMER.S193996

**Published:** 2019-05-03

**Authors:** Munirah Alsaleh, Thomas A Barbera, Helen L Reeves, Matthew E Cramp, Stephen Ryder, Hani Gabra, Kathryn Nash, Yi-Liang Shen, Elaine Holmes, Roger Williams, Simon D Taylor-Robinson

**Affiliations:** 1 Division of Surgery and Cancer, Imperial College London, London; 2 Northern Institute for Cancer Research, Medical School, University of Newcastle, Newcastle upon Tyne, UK; 3 Liver Unit, Derriford Hospital, Plymouth, Devon , UK; 4 Nottingham Digestive Diseases Centre, University of Nottingham, Nottingham, UK; 5 NIHR Biomedical Research Unit, Nottingham University Hospitals NHS Trust, Queen’s Medical Centre, Nottingham, UK; 6 Early Clinical Development, IMED Biotech Unit, AstraZeneca, Cambridge, UK; 7 Liver Unit, Southampton General Hospital, Southampton, Hampshire, UK; 8 Department of Radiation Oncology, Chang Gung Memorial Hospital and Chang Gung University, Taoyuan, Taiwan; 9 Institute of Hepatology, UK

**Keywords:** cholangiocarcinoma, metabolomics, diagnostic biomarkers

## Abstract

**Background:** Outside South-East Asia, most cases of cholangiocarcinoma (CCA) have an obscure etiology. There is often diagnostic uncertainty. Metabolomics using ultraperformance liquid chromatography mass spectrometry (UPLC-MS) offers the portent to distinguish disease-specific metabolic signatures. We aimed to define such a urinary metabolic signature in a patient cohort with sporadic CCA and investigate whether there were characteristic differences from those in patients with hepatocellular carcinoma (HCC), metastatic secondary liver cancer, pancreatic cancer and ovarian cancer (OCA).

**Methods:** Spot urine specimens were obtained from 211 subjects in seven participating centers across the UK. Samples were collected from healthy controls and from patients with benign hepatic disease (gallstone, biliary strictures, sphincter of Oddi dysfunction and viral hepatitis) and patients with malignant conditions (HCC, pancreatic cancer, OCA and metastatic cancer in the liver). The spectral metabolite proﬁles were generated using a UPLC-MS detector and data were analyzed using multivariate and univariate statistical analyses.

**Results:** The greatest class differences were seen between the metabolic proﬁles of disease-free controls compared to individuals with CCA with altered acylcarnitine, bile acid and purine levels. Individuals with benign strictures showed comparable urine proﬁles to patients with malignant bile duct lesions. The metabolic signatures of patients with bile duct tumors were distinguishable from patients with hepatocellular and ovarian tumors, but no difference was observed between CCA cases and patients with pancreatic cancer or hepatic secondary metastases.

**Conclusion:** CCA causes subtle but detectable changes in the urine metabolic proﬁles. The ﬁndings point toward potential applications of metabonomics in early tumor detection. However, it is key to utilize both global and targeted metabonomics in a larger cohort for in-depth characterization of the urine metabolome in hepato-pancreato-biliary disease.

## Introduction

Cholangiocarcinoma (CCA) is a devastating malignancy. Tumors commonly present late in the course of the disease and with the exception of liver fluke-associated CCA in South-East Asia are sporadic in most cases.^1^ The commonest known risk factor of CCA in developed countries is primary sclerosing cholangitis (PSC).^1^ In England and Wales, the incidence of CCA has increased year on year.^2^

**Figure 7:**
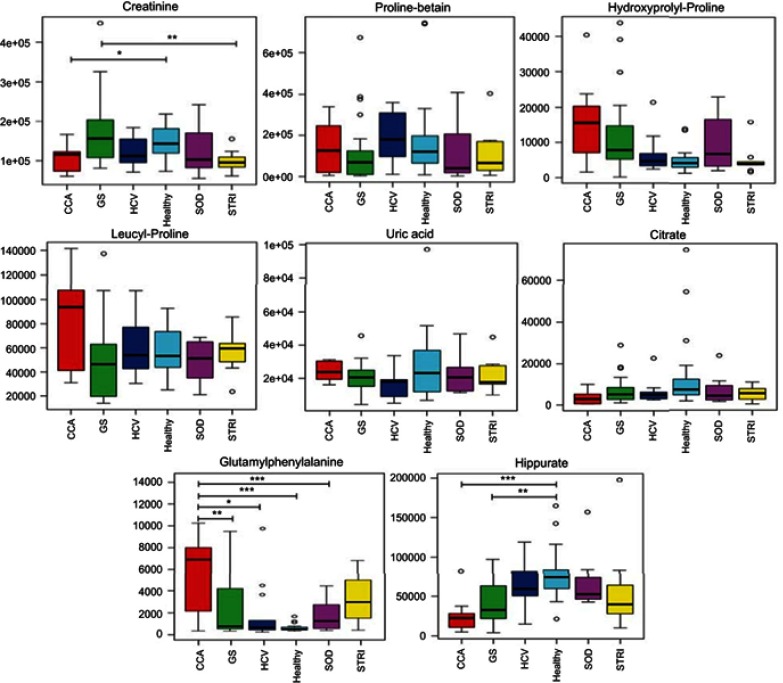
Box and whisker of compounds different between cholangiocarcinoma (CCA) cases and benign controls: ESI+ metabolites. *p 0.05, **p 0.01 and ***p 0.001. *Y*-axis= relative intensity. **Abbreviations:** CCA, cholangiocarcinoma; GS, gallstones; SOD, sphincter of Oddi dysfunction; STRI, strictures; HCV, noncirrhotic hepatitis C.

**Figure 8:**
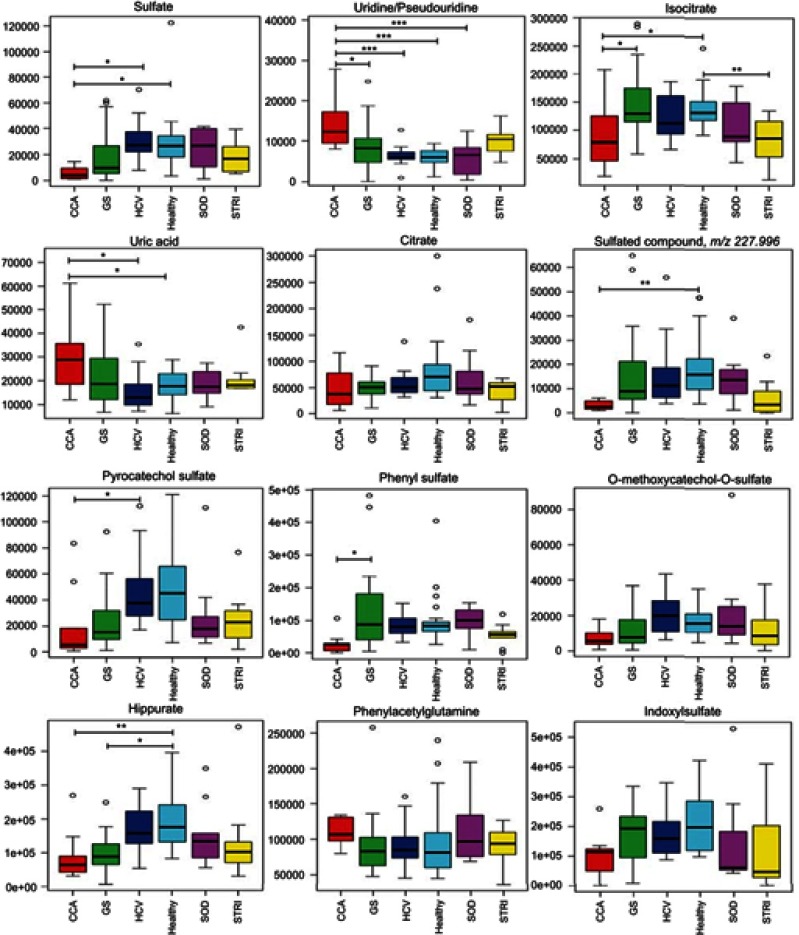
Box and whisker plots of compounds different between cholangiocarcinoma (CCA) cases and benign controls: ESI*−* metabolites. *p 0.05, **p 0.01 and ***p 0.001. *Y*-axis= relative intensity. **Abbreviations:** CCA, cholangiocarcinoma; GS, gallstones; SOD, sphincter of Oddi dysfunction; STRI, strictures; HCV, noncirrhotic hepatitis C.

Given the diagnostic uncertainty that surrounds most CCA cases, a metabolomic approach might shed light not only on disease pathogenesis in CCA, but also potentially provide information for diagnostic, predictive or prognostic use. Finding prognostic and diagnostic markers for CCA is urgent for liver ﬂuke endemic regions, particularly for Thailand, where CCA is a major health problem, but it is also important for sporadic CCA, where the disease is fatal in majority of the cases.^3^ Due to the rarity of CCA in Western countries, it is difﬁcult to acquire a sufﬁcient number of samples to robustly identify and validate metabonomics-based molecular markers using large-scale studies. We have thus collected samples across the UK to build up numbers in partnership with the UK Clinical Research Network.

The primary objective of the current study was to evaluate differences between the urinary metabolite proﬁles of CCA cases, compared to healthy participants, patients with benign hepatic disease (gallstones, strictures, sphincter of Oddi dysfunction [SOD] and viral hepatitis) and patients with malignant conditions (hepatocellular, pancreatic and metastatic cancers). Ovarian cancer (OCA) urine samples were included as nonhepatic malignancy controls, as these tumors are of a completely different cell line and we hypothesized that they would have a very distinct metabolic profile from those of a hepatobiliary or pancreatic origin.

## Methodology

### Patient and healthy volunteer recruitment

Urine samples were collected from seven participating UK clinical liver centers in London, Manchester, Newcastle, Nottingham, Plymouth and Southampton and transported frozen to the Hepatology Biobank at St. Mary’s Hospital, London, UK. Sequentially presenting potential participants were identiﬁed and recruited by their clinician from both the in-patient or out-patient populations.

Inclusion criteria:

- Established histological and/or imaging diagnosis of any hepato-pancreato-biliary malignancy, including:
  - CCA;
  - hepatocellular carcinoma (HCC);
  - pancreatic carcinoma;
  - liver metastases (any primary source).

- Established histological diagnosis of OCA, included as a malignant, nonhepatobiliary control.

- Established diagnosis of any benign hepato-pancreato-biliary condition, including:
  - gallstone disease;
  - PSC;
  - SOD;
  - pre-cirrhotic hepatitis C infection
- Matched control individuals with no hepatic-pancreato-biliary condition.

Healthy volunteers were sought from among visitors to the hospital, staff and students. After participants provided written informed consent, they were assessed at baseline for demographic data, medical history, drug history and dietary history. Ethical approval was obtained from Imperial College London REC, London, UK (REC Reference 09/H0712/82). This complied with the precepts set out in the 1975 Declaration of Helsinki on Human Rights.

### Sample processing

A fasting urine specimen of 20 mL was collected from each participant. Urine specimens were kept on ice or in +4°C fridge while awaiting processing. Samples were centrifuged at 4°C at 1,000 g for 10 mins and then 2 mL of urine aliquots was transferred into 6 Eppendorf tubes (Eppendorf Ltd, Stevenage, UK), of which one was used for the current study. Urine samples were transported on dry ice to the South Kensington campus, Imperial College London for MS analysis.

### Quality control

Quality control (QC) samples were prepared by pooling 50 μL of each urine sample into a Falcon tube (Sigma-Aldrich, Dorset, UK). A 200 μL aliquot was then transferred to an analytical MS well plate to enable acquisition of a QC spectrum every 10 samples.

### Chromatographic conditions

The sample spectra were acquired using an ACQUITY*^TM^* ultraperformance liquid chromatography mass spectrometry (UPLC) system (Waters Ltd. Elstree, UK), coupled to an LCT Premier™ mass spectrometer (Waters MS Technologies, Ltd., Manchester, UK). RP-UPLC-MS was performed with electrospray ionization (ESI) in both positive and negative modes. The conditions were optimized using the QC samples in terms of peak shape, reproducibility and retention time.

### Tandem mass spectrometry

Tandem mass spectrometry (MS/MS) analysis was performed using a quadrupole time-of-ﬂight (TOF) Premie™ instrument (Waters MS Technologies). Collision-induced dissociation (CID) experiments of the QC sample were performed for structural elucidation of detected ions in each ionization mode. This was conducted subsequent to the original proﬁling run to save time and limit analytical variations in retention time and performance that can occur when returning to the instrument for CID analysis.

Two complementary MS/MS acquisition modes were used to ensure sufﬁcient MS/MS coverage of ions of interest, data-dependent acquisition (DDA) and acquisition with no precursor ion selection, or data-independent acquisition (MS*^E^*). The DDA experiment was set to switch automatically from the MS to MS/MS mode using data-dependent criteria. It triggered MS/MS on the most abundant ions in each MS scan and provided fragments, which were speciﬁcally attributed to the precursor ion. In MS*^E^* mode, eluting peaks were subjected to both high and low collision energies in the collision cell of the mass spectrometer, with no prior precursor ion selection.^4^

### Metabolite assignment veriﬁcation

The molecular mass, retention time and fragmentation spectrum of the discriminant features were compared against online spectral libraries such as HMDB (www.hmdb.ca)^5^ and METLIN (https://metlin.scripps.edu).^6^ Metabolites were classiﬁed as either:

1) identiﬁed compounds conﬁrmed with an authentic standard; 2) putatively annotated compounds (such as those based upon fragmentation pattern and/or spectral similarity with spectral databases); 3) putatively identiﬁed to match a certain chemical class (such as those based on spectral similarity to known compounds of a chemical class); or 4) as unknown compounds.

### Preprocessing

The raw LC-MS data ﬁles were converted to CDV format by MassLynx*^TM^* version 4.1 application manager (Waters Corporation, Milford, CT, USA) and then imported into R Project version 3.1.0 (The R Foundation for Statistical Computing, 2014) for preprocessing using XCMS package version 2.14. (Bioconductor). Computational scripts written in-house were applied to: 1) ﬁlter and identify peaks; 2) correct for retention time drift; 3) match peaks across samples; and 4) ﬁll in missing peaks.

### Statistical analysis

SIMCA-P+ version 13.0.2 (Umetrics, Umeå, Sweden) was used for multivariate statistical analysis of the processed data. Initial analysis was performed using unsupervised principal component analysis (PCA) to explore variation in the dataset and examine clustering patterns or trends in the dataset, based on metabolic proﬁle similarities or differences. Following PCA, orthogonal projections to latent structures discriminant analysis (OPLS-DA) was performed to maximize separation between predeﬁned sample classes in order to view discriminatory features. Feature selection was based on the variable importance in projection (VIP) coefﬁcients, which allow the X variables to be classiﬁed according to their explanatory power of Y (class information). Features with high VIP value, >1, were found to be the most relevant for explaining Y class information. The top 30 features were selected and identiﬁed for each model.

Validating multivariate models is essential to avoid overﬁtting the data. The model statistics, R^2^X, Q^2^Y, permutation test and CV-ANOVA *p*-value, were used to evaluate the model’s robustness. Permutation testing (with 100 permutations) was calculated for every OPLS-DA model using SIMCA-P+ version 13.0.2 (Umetrics). Univariate signiﬁcance tests were then performed on the selected features using ANOVA with post-hoc testing (Tukey’s HSD), which is designed to account for multiple comparisons.

### Correlation with hierarchical clustering order

R Project version 3.1.0 (The R Foundation for Statistical Computing, 2014) using corrplot package version 0.77 (CRAN) was used to perform hierarchical cluster analysis of Spearman’s correlation coefﬁcient matrix. The cluster analysis was used to investigate correlations among the identiﬁed biochemical components. The correlation matrix was represented as a heatmap with rows and columns ordered according to a hierarchical clustering analysis. Hierarchical clustering is an unsupervised method. No class information was given to calculate the model, which was found to be suitable for exploratory data analysis. Positively and negatively correlated analytes were displayed in blue and red colors, respectively. A circle was used to represent correlations between pairs of compounds. The circle diameter and color intensity were proportional to the correlation coefﬁcients and indicate statistically signiﬁcant correlations (*<*0.05). The circle diameter and color intensity were proportional to the correlation coefﬁcients.

## Results

### Demographics, clinical data and cohort description

A total of 211 subjects provided urine samples for analysis using global LC-MS metabolic proﬁling. Table 1 shows the demographics and clinical description of the study cohort. Overall, the samples were categorized into 3 groups: healthy controls, participants with benign hepatic conditions (including SOD, strictures, gallstones and viral noncirrhotic hepatitis C [HCV]) and participants with malignant tumors (including CCA, HCC, pancreatic cancer, OCA and liver metastases cases). Of the 10 participants with CCA, 4 were diagnosed with perihilar CCA, 2 with distal CCA, 1 case with intrahepatic CCA and 3 cases with unknown tumor origin. Healthy controls (n=22) were younger than those with both benign and malignant hepatic conditions. With respect to gender, the HCC group comprised mostly of men (85.7%), followed by the pancreatic cancer patient group with 71.4% male participants. The participants’ racial background was diverse, but they were mostly white European.

**Table 1:** Demographics of study population

Characteristic	Benign					Malignant				
Healthy	SOD	GS	STRI	HCV	CCA	HCC	PCA	OCA	METS
Participants, n	22	8	22	9	14	10	45	22	48	11
Age (range), years	34 (24–58)	55 (30–74)	66 (24–81)	63 (45–74)	46 (29–56)	69 (57–78)	68 (48–82)	68 (51–81)	64 (45–88)	64 (38–78)
Male, %	45.4	37.5	31.8	55.5	35.7	44.4	85.7	71.4	0	63.6
BMI, kg/m^2^	25 (19.5–39)	n/a	n/a	25 (20–30)*	26 (20–32)	25 (21–27)*	30 (17–50)	29 (20–76)	n/a	26 (20–29.5)
Serum biochemistry										
Bilirubin, μmol/L	n/a	10.1 (4–21)	26 (5–192)	40 (10–189)	10 (3–18)	95 (20–375)	31 (4–338)	108 (7–411)	8.2 (2–30)	103 (6–443)
ALT, IU/L	n/a	53 (17–99)	79.3 (8–425)	121 (23–479)	84.9 (21–298)	181.3 (37–389)	45.8 (4–130)	155.3 (15–734)	17.1 (6–64)	98.6 (15–295)
ALP, IU/L	n/a	94 (20–184)	170 (45–376)	182(60–454)	76 (43–146)	594 (159–1242)	144 (14–550)	471 (51–1664)	85.5 (12–177)	643 (46–1905)
Albumin, g/L	n/a	27.6 (1–41)	34.3 (24–43)	37.5 (31–41)*	40.1 (34–46)	29.8 (20–49)	36 (20–46)	30.3 (17–47)	33.7 (16–44)	31.7 (18–48)
Urea, mmol/L	n/a	26.5 (3.2–109)	5.2 (2.1–14.7)	7.3(3.9–16.7)*	4.7 (2.6–6.1)*	5.2 (1.9–16)	5.5 (2.5–10.7)	4.6 (2.5–7.7)*	4.7 (1.8–18.3)	5.7 (2.6–12.6)*
Creatinine, μmol/L	n/a	66 (54–83)*	72 (47–137)	71 (3.3–131)	73 (58–93)	77 (53–146)	82 (49–204)	67 (53–101)*	71(46–132)	84 (52–121)*
Race										
White	15	7	19	7	12	8	42	14	13	9
Black			1		1	1			1	
Asian	2		2	1				3	2	1
Asian-Indian	3							2	4	
Hispanic		1					1			
Middle Eastern	2				1		1	1	3	1
Not available				1		1	1	2	25	

**Note**: *Missing data.

**Abbreviations**: ALT, alanine transaminase; ALP, alkaline phosphatase; CCA, cholangiocarcinoma; GS, gallstones; HCC, hepatocellular carcinoma; HCV, noncirrhotic hepatitis C; METS, metastatic cancer of the liver; OCA, ovarian cancer; PCA, pancreatic cancer; SOD, sphincter of Oddi dysfunction; STRI, strictures; n/a, not available.

### CCA patients vs benign liver disease cases

**Figure 1:**
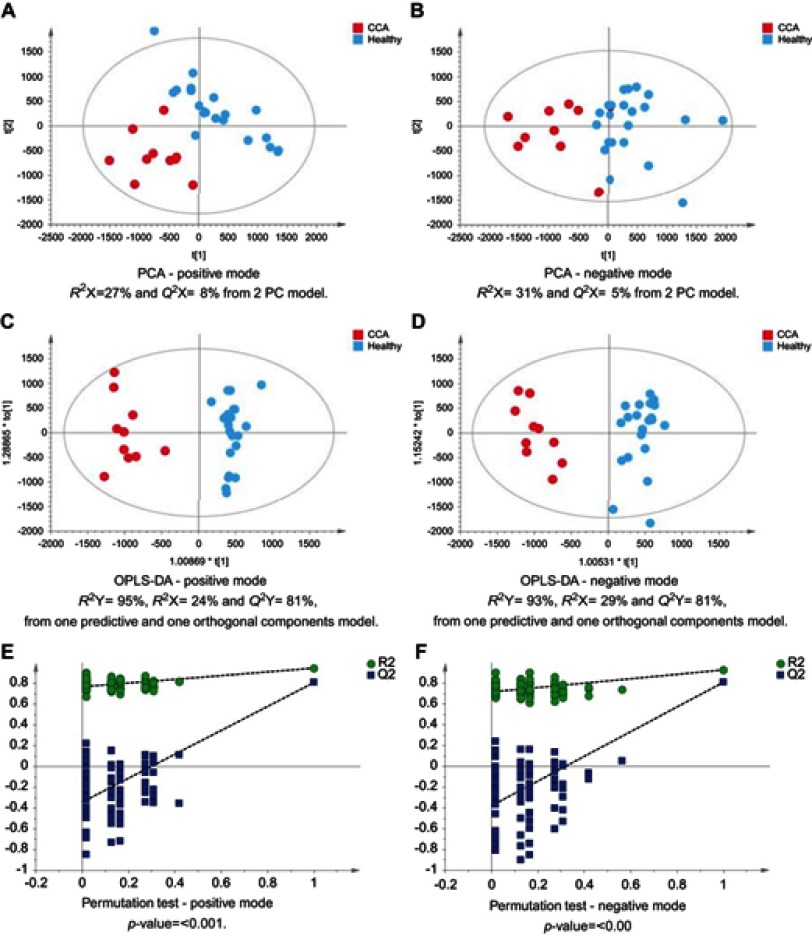
CCA patients vs healthy controls. PCA scores plots for (**A**) positive and (**B**) negative ion mode data of CCA patients and healthy controls. OPLS-DA scores plots showing group separation for both (**C**) positive and (**D**) negative ion mode data and the corresponding permutation tests for (**E**) positive and (**F**) negative ion mode data.**Abbreviations:** CCA, cholangiocarcinoma; PCA, principal component analysis; OPLS-DA, orthogonal projections to latent structures discriminant analysis.

**Figure 2:**
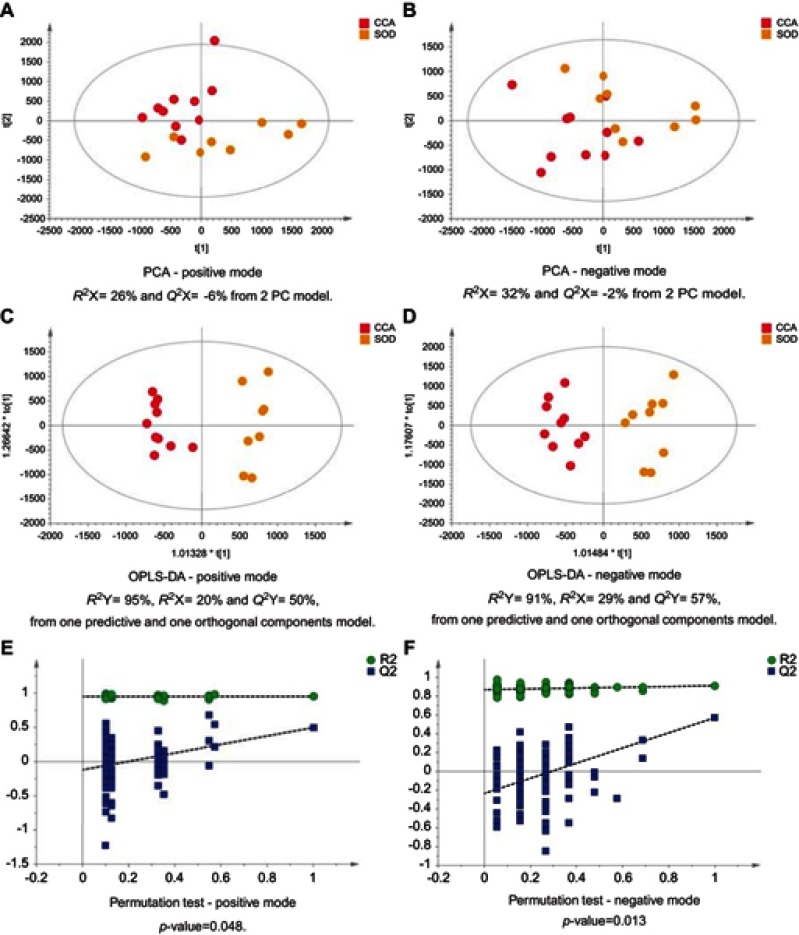
CCA patients vs patients with SOD. PCA scores plots for (**A**) positive and (**B**) negative ion mode data of CCA patients and SOD cases. OPLS-DA scores plots showing group separation for both (**C**) positive and (**D**) negative ion mode data and the corresponding permutation tests for (**E**) positive and (**F**) negative ion mode data. **Abbreviations:** CCA, cholangiocarcinoma; SOD, sphincter of Oddi dystfunction; PCA, principal component analysis; OPLA-DA, orthogonal projections to latent structures discriminant analysis.

**Figure 3:**
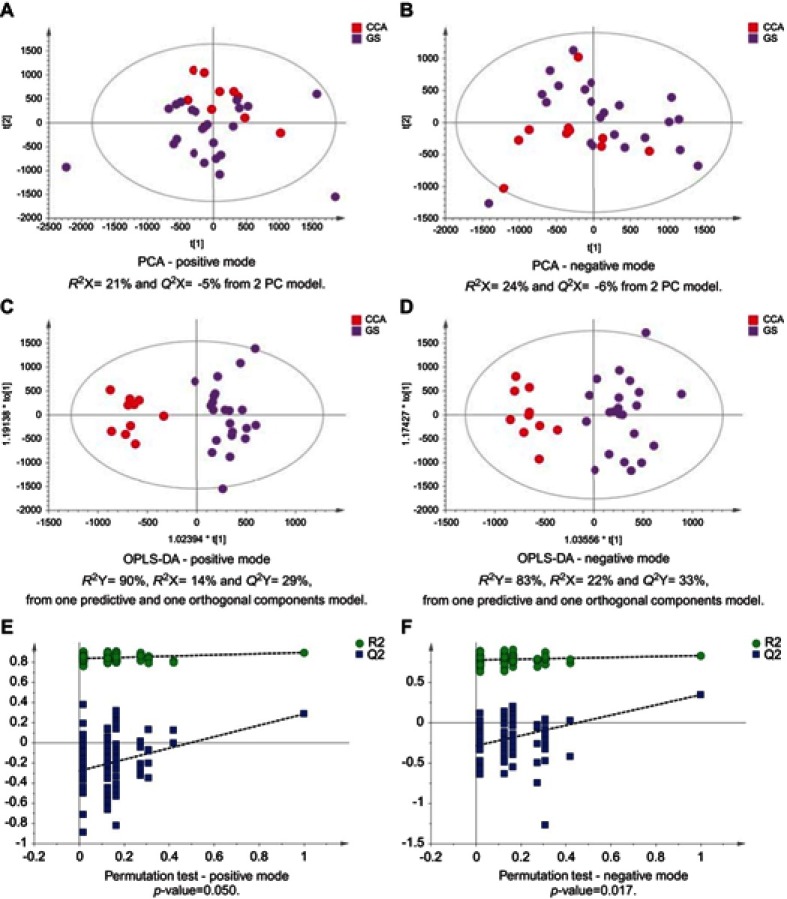
CCA patients vs patients with gallstones. PCA scores plots for (**A**) positive and (**B**) negative ion mode data of CCA patients and gallstones cases. OPLS-DA scores plots showing group separation for both (**C**) positive and (**D**) negative ion mode data and the corresponding permutation tests for (**E**) positive and (**F**) negative ion mode data. **Abbreviations:** CCA, cholangiocarcinoma; PCA, principal component analysis; OPLS-DA, orthogonal projections to latent structures discriminant analysis.

**Figure 4:**
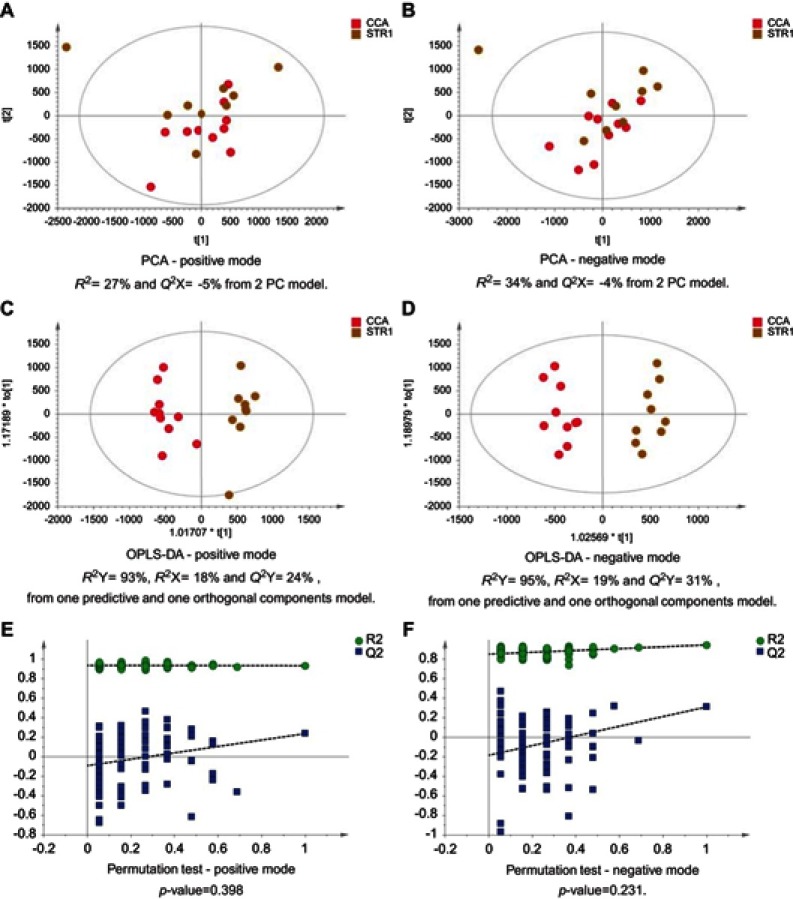
CCA patients vs biliary strictures patients. PCA scores plots for (**A**) positive and (**B**) negative ion mode data of CCA patients and biliary strictures patients. OPLS-DA scores plots showing group separation for both (**C**) positive and (**D**) negative ion mode data and the corresponding permutation tests for (**E**) positive and (**F**) negative ion mode data. **Abbreviations:** CCA, cholangiocarcinoma; PCA, principal component analysis; OPLS-DA, orthogonal projections to latent structures discriminant analysis.

**Figure 5:**
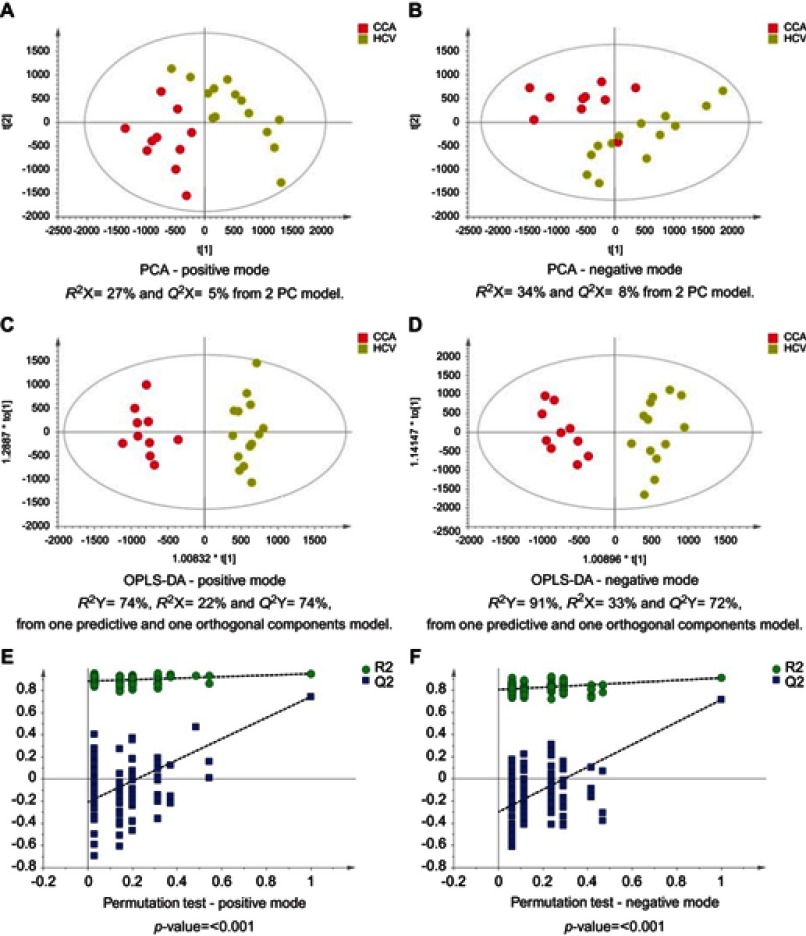
CCA vs noncirrhotic HCV patients. PCA scores plots for (**A**) positive and (**B**) negative ion mode data of CCA patients and HCV patients. OPLS-DA scores plots showing group separation for both (**C**) positive and (**D**) negative ion mode data and the corresponding permutation tests for (**E**) positive and (**F**) negative ion mode data. **Abbreviations:** CCA, cholangiocarcinoma; HCV, noncirrhotic hepatitis C; PCA, principal component analysis; OPLS-DA, orthogonal projections to latent structures discriminant analysis.

The urinary spectral data from CCA patients were compared to benign liver disease groups using OPLS-DA analysis (Figure 1–5). The most discriminant OPLS-DA model was the one between the urinary MS spectral proﬁle from CCA cases, compared to healthy participants (Figure 1). Similarly, profiles from patients with SOD and gallstones were distinguishable from CCA patients (Figures 2 and 3). The urinary spectral data from participants with benign strictures, including PSC cases, were poorly differentiated from CCA patients (Figure 4). The model comparing CCA patients versus a disease control group – those with pre-cirrhotic chronic hepatitis C infection – did show proﬁle separation (Figure 5).

### Altered urinary metabolites between CCA patients and benign cases

**Figure 6:**
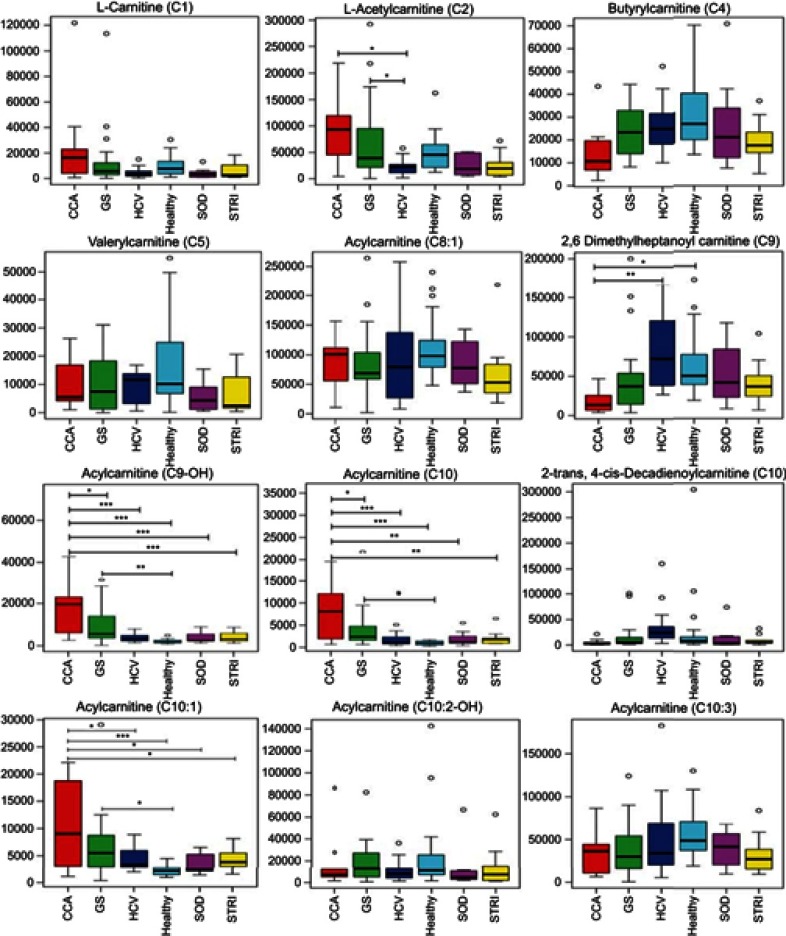
Box and whisker plots of compounds different between cholangiocarcinoma (CCA) cases and benign controls–carnitine species. *p 0.05, **p 0.01 and ***p 0.001. Y-axis= relative intensity. **Abbreviations:** CCA, cholangiocarcinoma; GS, gallstones; SOD, sphincter of Oddi dysfunction; STRI, strictures; HCV, noncirrhotic hepatitis C.

**Figure 9:**
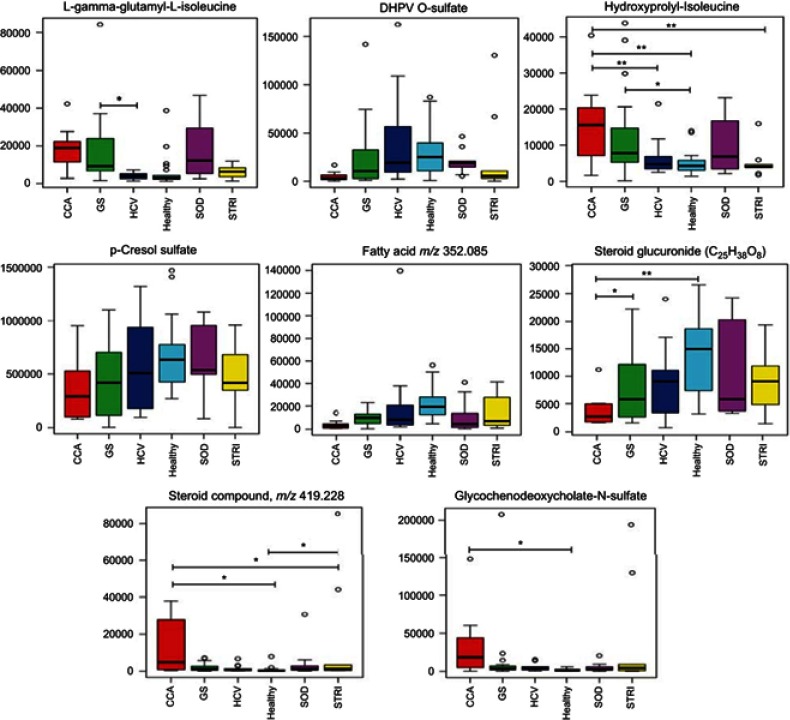
Box and whisker plots of compounds different between cholangiocarcinoma (CCA) cases and benign controls: ESI*−* metabolites. *p 0.05, **p 0.01 and ***p 0.001. *Y*-axis= relative intensity. **Abbreviations:** CCA, cholangiocarcinoma; GS, gallstones; SOD, sphincter of Oddi dysfunction; STRI, strictures; HCV, noncirrhotic hepatitis C.

The urinary molecules inﬂuencing the OPLS-DA model separation between CCA cases and benign disease conditions (SOD, HCV and gallstones) were similar to those identiﬁed from the model comparing CCA to healthy participants. Therefore, to avoid repetition of data, Tables 2 and 3 list the metabolites driving the separation between the urinary metabolic proﬁles from CCA patients compared to healthy controls. Differences in these metabolic features between the groups were then evaluated using ANOVA with post hoc testing. The relevant metabolites are further illustrated in Figures 6–9.

**Table 2:** Altered metabolites between cholangiocarcinoma patients compared to healthy controls – positive mode

*m*/*z*	RT	Tentative assignment	Adduct	VIP	Trend	*p*-value^†^	FC	Identiﬁcation*
162.113	0.49	L-carnitine (C1)	M+H	4.3	↑	NS	2.62	a
114.066	0.51	Creatinine	M+H	5.9	↓	0.043	−1.4	a
144.102	0.58	Proline betaine	M+H	4.5	↓	NS	−0.67	b
229.118	0.68	Hydroxyprolyl-proline	M+H	3.3	↑	NS	1.94	b
204.125	0.82	L-Acetylcarnitine (C2)	M+H	7.7	↑	NS	1.87	a
229.155	0.98	Leucyl-proline	M+H	5.2	↑	NS	1.45	b
169.036	1.03	Uric acid	M+H	3	↓	NS	−0.9	a
215.016	1.06	Citrate	M+Na	3.2	↓	NS	−3.58	a
188.071	2.51	Unknown	-	4.8	↑	NS	2.22	d
232.155	2.84	Butyrylcarnitine (C4)	M+H	4.4	↓	0.023	−2.22	a
296.15	3.17	Unidentiﬁed acylcarnitine	M+H	3.3	↑	NS	0.14	a
295.13	3.54	Glutamylphenylalanine	M+H	3.2	↑	<0.0001	12.194	b
246.17	3.57	Valerylcarnitine (C5)	M+H	3.4	↓	NS	−1.78	a
318.191	3.78	Acylcarnitine (C9-OH)	M+H	6.3	↑	<0.0001	9.25	b
178.05	3.8	Hippurate	M+H	8.9	↓	<0.0001	−3	a
344.207	4.4	Acylcarnitine (C10:1)	M+H	3.1	↑	0.001	4.43	b
346.223	4.57	Acylcarnitine (C10)	M+H	3.9	↑	0.0002	7.88	b
170.061	4.81	Unknown	-	5.2	↓	0.001	−3.41	d
286.201	4.92	Acylcarnitine (C8:1)	M+H	6.4	↓	NS	−0.79	b
277.142	5.12	Unknown	M+H	3.5	↑	NS	0.1	b
312.217	5.16	2-trans,4-cis-Decadienoylcarnitine (C10)	M+H	3.9	↓	NS	−5.78	b
310.202	5.19	Acylcarnitine (C10:3)	M+H	5.2	↓	NS	−1.57	b
310.202	5.35	Acylcarnitine (C10:3)	M+H	5.4	↓	0.021	−1.85	b
302.233	5.55	2,6-Dimethylheptanoyl carnitine (C9)	M+H	7.6	↓	0.001	3.65	b
314.233	5.78	Decenoylcarnitine (C10:1)	M+H	3.1	↓	NS	1.62	b
328.248	6.11	Acylcarnitine (C10:2-OH)	M+H	4	↓	NS	1.43	b

**Notes:** *Level of metabolite identification: (a) identified compound; (b) putitatively annotated compound; (c) putitatively characterized compound class; and (d) unknown. ^†^False discovery rate (FDR) adjusted *p*-value (or *q*-value).

**Abbreviations:** FC, fold change; RT, retention time; VIP, variable importance in projection score.

**Table 3:** Altered metabolites between cholangiocarcinoma patients compared to healthy controls – negative mode

*m*/*z*	RT	Tentative assignment	Adduct	VIP	Trend	*p*-value^†^	FC	Identiﬁcation*
96.962	0.60	Sulfate	M-H	3.8	↓	0.025	−4.83	b
243.061	0.85	Uridine/pseudouridine	M-H	3.1	↑	0.0002	2.24	b
191.018	0.94	Isocitrate	M-H	5.8	↓	0.023	−1.58	b
167.02	1.03	Uric acid	M-H	3.1	↑	0.011	1.7	a
191.018	1.06	Citrate	M-H	4.7	↓	NS	−1.93	a
227.996	2.65	Sulfated compound		3.2	↓	0.005	−6.12	d
188.985	3.13	Pyrocatechol sulfate	M-H	4.5	↓	0.03	−2.66	b
172.991	3.45	Phenyl sulfate	M-H	6.8	↓	0.039	−3.44	b
203.001	3.75	O-methoxycatechol-O-sulfate	M-H	3.0	↓	0.014	−2.45	b
178.049	3.80	Hippurate	M-H	8.5	↓	0.006	−2.2	a
263.102	3.84	Phenylacetylglutamine	M-H	4.4	↑	NS	1.11	b
212.001	3.87	Indoxylsulfate	M-H	9.1	↓	0.021	−1.93	a
241.118	3.92	L-gamma-glutamyl-L-isoleucine	M-H2O-H	3.2	↑	0.008	3.14	b
287.022	4.13	DHPV O-sulfate^‡^	M-H	4.2	↓	0.018	−6.32	b
243.134	4.18	Hydroxyprolyl-isoleucine	M-H	2.9	↑	0.002	3.24	b
187.005	4.41	*p*-cresol sulfate	M-H	17	↓	0.051	−1.85	b
352.085	5.88	Fatty acid		3.6	↓	0.004	−5.31	d
465.248	6.37	Steroid glucuronide (C25H38O8)	M-H	3.0	↓	0.001	3.9	b
419.228	6.48	Steroid compound		3.1	↑	0.005	15.3	d
528.263	6.76	Glycochenodeoxycholate-N-sulfate	M-H	4.4	↑	0.011	18.3	b

**Notes**: *Level of metabolite identification: (a) identified compound; (b) putatively annotated compound; (c) putatively characterized compound class; and (d) unknown. ^†^False discovery rate (FDR) adjusted *p*-value (or *q*-value). ^‡^5ʹ-(3ʹ,4ʹ-dihydroxyphenyl)-gamma-valerolactone sulfate.

**Abbreviations**: FC, fold change; RT, retention time; VIP, variable importance in projection score.

**Figure 10:**
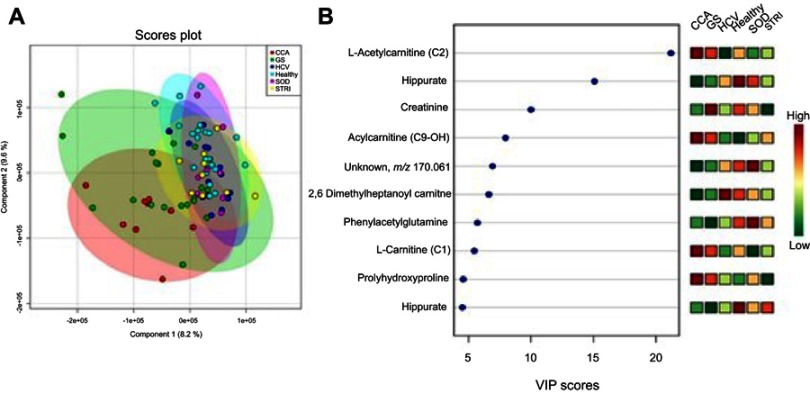
Urine metabolites associated with the metabolic signatures of the six distinct disease conditions – positive mode. (**A**) Scores plot of the principal component analysis (PCA) analysis, *R*2Y=41% and *Q*2Y=16% from 2 PC model, *p*-value=0.01 from permutation test and (**B**) important metabolites selected on the basis of VIP score. **Abbreviations:** CCA, cholangiocarcinoma; GS, gallstones, HCV, noncirrhotic hepatitis C; SOD, sphincter of Oddi dysfunction; STRI, strictures.

Multigroup PLS-DA analysis was calculated to further assess the molecular panel underpinning the urinary metabolic differences between the six distinct conditions. The statistical model generated scores plot and a corresponding plot which rank metabolites, based on their VIP scores. Based on the crossvalidation statistics, differences in the urinary metabolic signature were identiﬁed in the ESI^+^spectral data (Figure 10), but not the ESI*^−^* spectral data.

### CCA patients vs other cancers

**Figure 11:**
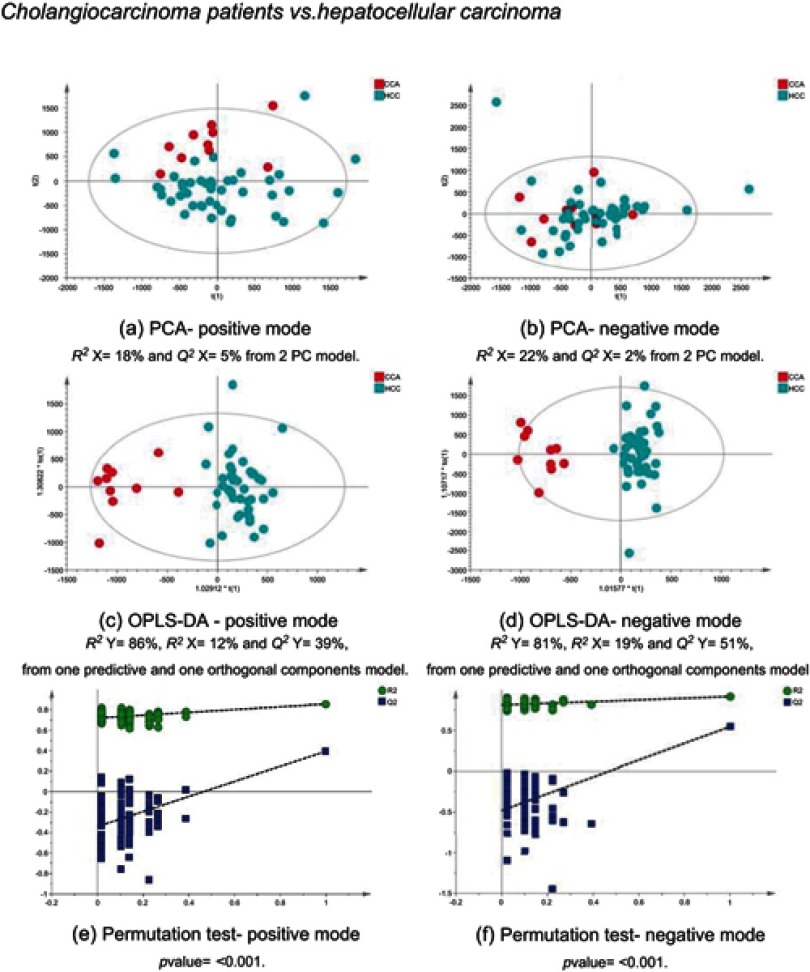
CCA patients vs HCC patients. PCA scores plots for (**A**) positive and (**B**) negative ion mode data of CCA patients and HCC patients. OPLS-DA scores plots showing group separation for both (**C**) positive and (**D**) negative ion mode data and the corresponding permutation tests for (**E**) positive and (**F**) negative ion mode data. **Abbreviations:** CCA, cholangiocarcinoma; HCC, hepatocellular carcinoma; PCA, principal component analysis; OPLA-DA, orthogonal projections to latent structures discriminant analysis.

**Figure 12:**
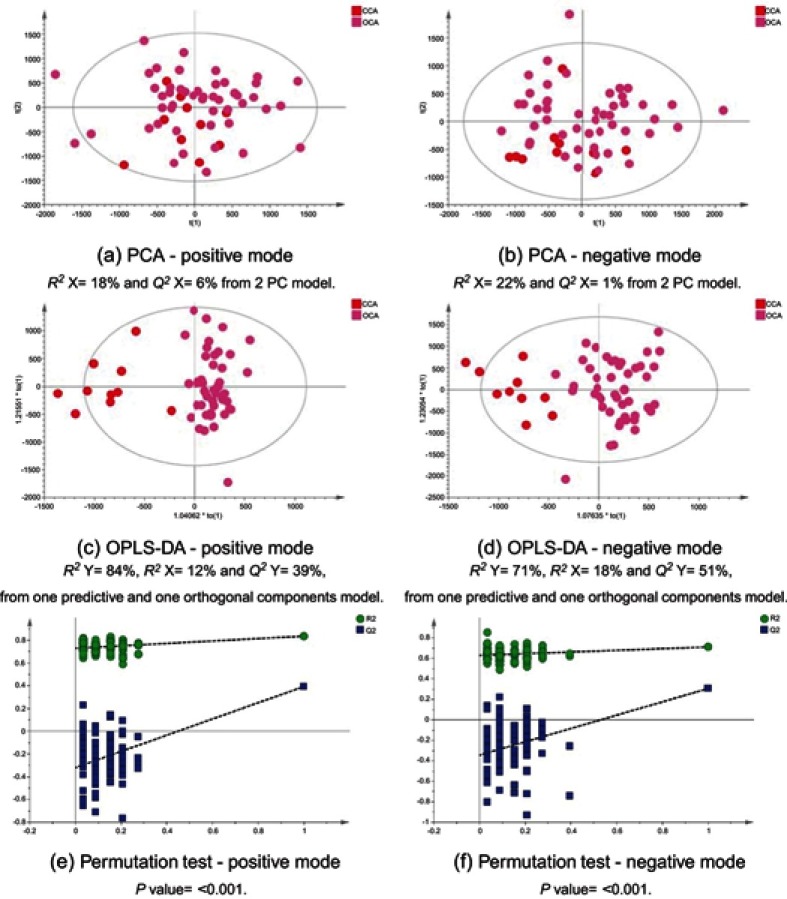
CCA patients vs ovarian cancer. PCA scores plots for (**A**) positive and (**B**) negative ion mode data of CCA patients and OCA patients. OPLS-DA scores plots showing group separation for both (**C**) positive and (**D**) negative ion mode data and the corresponding permutation tests for (**E**) positive and (**F**) negative ion mode data. **Abbreviations:** PCA, principal component analysis; CCA, cholangiocarcinoma; OCA, ovarian cancer; OPLS-DA, orthogonal projections to latent structures discriminant analysis.

The MS data acquired using urine samples from CCA patients were compared to those acquired from patients with malignant pathologies. Patients with CCA showed a distinguishable urinary metabolic proﬁle from patients with hepatocellular (Figure 11) and ovarian tumors (Figure 12), but no difference was observed between CCA cases and patients with pancreatic and metastatic cancers. The urine proﬁles from patients with pancreatic tumors also did not differ from metastatic cases.

## Discussion

The study identiﬁed a coherent metabolic pattern associated with bile duct tumors. This molecular pattern was similar to the one associated with participants with pancreatic and metastatic cancers, yet distinct from individuals with hepatocellular and ovarian tumors. The urine metabolic proﬁles from CCA patients were also distinguishable from patients with benign liver pathologies (including SOD, gallstones and chronic noncirrhotic hepatitis C). However, the urinary metabolome from individuals with benign biliary strictures revealed a relatively similar underpinning metabolic process, compared to malignant strictures.

As expected, the best separation in the urinary metabolome was achieved between the spectral proﬁles from CCA patients and healthy controls. Interestingly, the model comparing the proﬁles of patients with CCA and benign strictures (including PSC cases) did not achieve statistical signiﬁcance. Yet, participants with benign strictures showed a distinct molecular pattern from healthy controls.

Metabolites driving the separation between the two classes (healthy vs benign biliary strictures) were relativity similar to those separating CCA and healthy controls with depletion in urinary acylcarnitine and creatinine associated with patients with biliary strictures. In the ESI*^−^*, a decrease in the levels of urinary p-cresol sulfate, indoxylsulfate, isocitrate and hippurate and a higher level of sulfated glycol-ursodeoxycholic acid was characteristic of the urinary metabotype of patients with bile duct strictures, compared to healthy controls. Only one molecule, isocitrate, achieved statistical signiﬁcance between patients with strictures and healthy controls.

The urinary metabolites altered between benign and malignant bile duct strictures were primarily related to acylcarnitine metabolism, where the levels of (C9-OH, C10 and C10:1) carnitines were signiﬁcantly downregulated in subjects with benign strictures.

Deﬁnitive diagnosis to distinguish benign from malignant lesions involves multiple tools including imaging, endoscopic investigations, tissue sampling and diagnostic surgery.^7^ Even after preoperative evaluation, biliary strictures can remain indeterminate.^8^ The serum metabolic signatures of PSC patients in US population has been proﬁled using LC and GC mass-spectroscopic detection techniques.^9^ Markers related to inﬂammatory status, as well as to perturbations in bile acid metabolism (elevations in cholesterol, unconjugated, conjugated and sulfated bile acid), lipid metabolism (elevations in fatty acids, ketone bodies and several acylcarnitines) and protein metabolism (depletion in dipeptides), were associated with the PSC patient’s metabolome, compared to healthy controls.^9^ The authors postulated that these changes reﬂect cholestatic liver state, perturbations in cholesterol homeostasis and mitochondrial dysfunction which are typical features associated with impaired fatty acid *β*-oxidation.

It is not clear why the urine metabolome of patients with benign biliary strictures was distinguishable from healthy controls, but not from those with malignant biliary tumors. These results may suggest that cellular turnover related to underlying biliary tract inflammation in benign or malignant cholestatic liver disease is likely to exhibit similar metabolic traits in the urine metabolome. However, it is difﬁcult to draw ﬁrm conclusions from these data due to the small sample size (CCA=10 participants and benign biliary strictures=9 participants).

In the current literature, metabolic phenotyping studies in hepatobiliary disease have been mainly focused on bile proﬁling. Differences in the biochemical composition of bile between CCA and patients with nonmalignant biliary disease (including various conditions such as SOD, PSC and gallstones) were distinguished between the two groups.^10^,^11^ One study examined bile from PSC (n=18) patients and from patients with CCA (n=16), but also included 11 who had other benign biliary conditions. The spectra of bile from CCA patients differed from the benign group in the levels of phosphatidylcholine, bile acids, lipids and cholesterol with sensitivity, speciﬁcity and accuracy of 88.9%, 87.1% and 87.8%, respectively.^12^

### Metabolic proﬁle discrimination between CCA and malignant disease groups

The metabolic proﬁle acquired using urine specimens from CCA patients was distinguishable from those with hepatic and ovarian malignancies, but not from individuals with metastatic and pancreatic tumors. Urinary acylcarnitine pattern and excess excretion of urinary bile acids were comparable between the two groups. The urine proﬁles from pancreatic cancer patients were not distinguishable from CCA and metastatic patients and also shared similarity, based on their urinary biochemical composition.

Pancreatic and bile duct tumors (particularly bile duct tumors that arise in the distal extrahepatic region) share a close anatomical relation, similar growth pattern and close phenotypic characteristics and are associated with poor prognosis, which may explain the similarity in their urine metabolome.^13^ In a recently published review on the subject by Schmuck et al, the authors discussed the various similar aspects between the two malignancies in terms of embryological, pathological, biological, clinical and surgical observations.^13^ For example, the most common mutations associated with CCA and pancreatic cancer are mutations in p53 and KRAS genes. Additionally, serum CA19–9 level, the most widely used tumor marker in pancreatobiliary diseases, is used as a biomarker for the clinical management of patients with CCA and pancreatic cancer. However, it is not possible to differentiate between benign, precursor lesions and malignant pancreatobiliary conditions using CA 19-9 levels alone.^14^ Schmuck et al postulated that the two malignancies should be regarded as a common tumor entity under a uniﬁed superfamily titled “tumours of the pancreatobiliary junction”.^13^

Metabolic dysfunctions in several molecular pathways implicated in carcinogenesis such as ketogenesis (increase in acetoacetate and decrease in citrate) were described to be associated with the nuclear magnetic resonance urine metabolome from pancreatic cancer patients.^15^ A number of candidate biomarkers in serum were proposed including bile acids, amino acids, nucleotides and fatty acids.^16^,^17^

Napoli et al investigated the spectral correlations, in the urine metabolic proﬁle from individuals with pancreatic cancer, with pathological staging and tumor anatomical localization. Patients with intermediate pancreatic carcinoma stage showed different urinary metabolome from those with advanced tumor stage. Also, a clear distinction was observed based on the anatomical location of pancreatic cancer, particularly between carcinoma of the uncinate process of the pancreas and pancreatic cancers of the body and head.^15^ Unlike CCA, pancreatic cancer is not rare: it is the fourth cause of cancer death in Europe.^18^ It has a rising predicted death trend in both genders, 4% in men and 5% in women between 2009 and 2015, corresponding to 85,300 total deaths in 2015.^18^

It is difﬁcult to draw a complete picture of the biological pathways implicated in pancreatobiliary conditions from this small dataset. To enable accurate metabonomic characterization of pancreatobiliary tumors, metabolic ﬁngerprinting using large sample size and more complete clinical information is needed. The possible application of metabolic proﬁling to differentiate different tumor localization and disease staging would helpfully provide a more comprehensive picture of tumor development and progression in the tumors of the pancreatobiliary junction.

Compared to HCC and OCA cases, urine from CCA patients showed a distinct pattern. A number of metabolites were signiﬁcantly different between the urine proﬁle from CCA patients, compared to HCC and OCA cases and may possibly serve as candidate biomarkers in CCA. These include acylcarnitine (C9:OH), uridine/pseudouridine, glucosamine-6-phosphate and certain bile acids. The key metabolic pathways most associated with cholangiocarcinogenesis are discussed below.

### Acylcarnitine metabolism

Several species of acylcarnitines were dysregulated in individuals with CCA, compared to control groups. An acylated carnitine compound, tentatively identiﬁed as acylcarnitine C9-OH, was signiﬁcantly greater abundance in CCA metabolic proﬁles compared to all groups, except patients with pancreatic and metastatic tumors. Multigroup PLS-DA identiﬁed carnitine-related metabolites (carnitine [C1], acetylcarnitine [C2] and acylcarnitine [C9-OH]) to be most associated with CCA cases, compared to nonmalignant conditions.

Elevated urinary concentrations of acylcarnitine metabolites were also observed in individuals with gallstones. ANOVA analysis showed that individuals with gallstones had signiﬁcantly greater levels of acylcarnitine metabolites (including C2, C9-OH, C10 and C10:1), with respect to healthy controls. Gallstone disease is a common disorder of the hepatobiliary system, characterized by the formation of gallstones (or cholelithiasis) in the gallbladder, common bile duct or hepatic bile duct. Cholelithiasis is a well-established risk factor for gallbladder carcinoma; it causes severe injury to the biliary mucosa which triggers a series of events starting with chronic inﬂammation to metaplasia, dysplasia, in situ carcinoma to invasive carcinoma.^19^

Gallbladder stone chemical composition is heterogeneous and the stone formation is aggravated by several lifestyle and environmental factors, such as low physical activity, high dietary fat intake, female gender and genetic predisposition.^20^ The presence of metabolic disorders including adiposity, hypertension, diabetes mellitus and lipid abnormalities is also associated with a higher prevalence of gallstone disease.^20^ Considering all these factors, in addition to the lack of comprehensive metabolic proﬁling studies on the disease, makes it difﬁcult to attempt to capture and characterize the urinary biochemical perturbation in individuals with cholelithiasis. Impaired metabolism of cholesterol, phospholipids, bilirubin and bile acids is known to be implicated in the pathogenesis of gallstone disease.^19^

### Metabolism of pyrimidine and purine nucleotides

Nucleotides, purines and pyrimidines are the nitrogenous bases of the genetic code.^21^ Nucleotides and their derivatives contribute to many biological processes such as cellular signaling, energy production and lipid and protein synthesis. Aberrant DNA replication and damage occur early during cellular carcinogenesis, in which imbalance of nucleotide metabolism, particularly deoxyribonucleotide triphosphates (dNTP), drives tumorigenesis and leads to nucleotide pool imbalance in tissue and biological ﬂuids.^22^ These nucleotide pool patterns may serve as a novel screening and diagnostic biomarkers for various human cancers.^22^ Increased concentrations of modiﬁed nucleotides (such as pseudouridine, 1-methyladenosine and 1-methylguanosine) in the urine of cancer patients are reﬂective of high whole-body RNA turnover or oxidative DNA damage.^23^

Nucleotide post-transcriptional chemical modiﬁcations, including methylation, hydroxylation, acetylation and uridine isomerization, are believed to play a key role in the translation of the genetic code, yet their exact biological function remains unclear. Ridine or pseudouridine, a structural C-glycoside isomer of the nucleoside uridine, was signiﬁcantly increased in CCA compared to all groups except individuals with strictures and pancreatic tumors. The pyrimidine nucleoside plays a crucial role in the synthesis of RNA, glycogen and biomembrane.^24^ Uridine is used clinically as a rescue agent that protects against 5-ﬂuorouracil toxicity.^25^ Medication history records showed that none of the individuals with CCA consumed 5-ﬂuorouracil or uridine-containing prodrugs.

The circulating plasma uridine level is tightly regulated in humans, yet several factors can result in elevated plasma uridine concentration, such as enhanced ATP consumption, enhanced uridine diphosphate (UDP)-glucose consumption via glycogenesis, increased urea synthesis and increased 5-phosphoribosyl-1-pyrophosphate.^24^ Ka et al demonstrated positive correlations between plasma uridine concentrations and the urinary excretions of urea, uric acid, uridine, uric acid clearance and purine intake in healthy males.^26^ The liver regulates and maintains the hepatic pools of uridine nucleotides via de novo uridine synthesis and degradation which is essential for the homoeostatic control of plasma uridine pools.^27^ An in vivo study evaluated uridine homeostasis in liver tissue and its impact on hepatic cellular function.^28^ The authors revealed that uridine administration suppresses fatty liver by modulating liver protein acetylation proﬁle and identiﬁed an association between uridine homeostasis, pyrimidine metabolism and liver lipid metabolism.^28^

We found an imbalance of purine metabolites in the urinary metabolome of CCA patients. Purine-based metabolites in this UK cohort showed increased uric acid, increased 7-methylguanine and decreased hypoxanthine. In the discriminant analysis applied to generate metabolite patterns as a method of disease identiﬁcation, uric acid, hypoxanthine and 7-methylguanine were all key metabolites responsible for the differences in metabolic signatures in CCA cases compared to the malignant controls.

### Bile acid metabolism

The greatest abundance of bile acid species was found in the urine metabolic proﬁles from individuals with pancreatobiliary tumors. It is difﬁcult to assess bile acid proﬁle in the bile duct and pancreatic carcinomas as they are frequently complicated with profound jaundice, which subsequently causes a marked increase in bile acid concentrations in both serum and urine.^29^,^30^ Nevertheless, elevation in bile acid species, such as tauroursodeoxycholic acid, taurocholic acid, deoxycholylglycine and cholylglycine, has been observed in the plasma MS metabolic proﬁles from nonjaundiced patients with early-stage pancreatic ductal adenocarcinoma.^31^ In a recent metabonomics study by Di Gangi et al, free plasma bile acid concentrations were signiﬁcantly decreased, whereas conjugated (glycine- and taurine-) cholic and chenodeoxycholic acids were signiﬁcantly increased in pancreatic cancer cases compared to disease-free controls.^16^ Cytotoxicity and cytoprotection by bile acids vary depending on their hydrophobic/hydrophilic properties. In jaundiced serum of pancreatic cancer cases, elevation in conjugated bile acids seems to exhibit a protective effect against pancreatic cell proliferation.^32^ Possible mechanisms of the inhibitory action of bile acids involve their cytotoxic potency which reduces pancreatic cancer cell line proliferation and results in structural damage.^32^ Little is known about the causative role in metabolic perturbations and oncogenic pathways preceding pancreatic cancer progression.^33^

In their review, Feng et al aimed to evaluate bile acids role in the etiology of pancreatic carcinogenesis. However, more questions are raised than have been answered; bile acids are not only involved in multiple risk factors known to be implicated in pancreatic cancer initiation, including obesity, diabetes, high-fat diet and gallstones, but they also exhibit biologically complex local tissue effects.^33^ They play a pathogenic and/or protective role which is complex to simulate the *ex vivo* environment.

## Conclusions

Primary data from this dataset identiﬁed multimolecular signatures of pancreatobiliary disease and illustrated the potential of MS-based metabolic proﬁling in generating a novel noninvasive diagnostic tool for tumor detection and may further understand disease mechanisms. Discriminant metabolites related to biliary stricturing conditions (both benign and CCA) showed a similar pattern. It was also not possible to differentiate between the metabolic proﬁles of patients with pancreatic cancer from CCA or from premalignant biliary strictures, but metabolic profiles of patients with HCC and OCAs were distinct from those with pancreatobiliary pathology.

It is important to highlight that its difﬁcult to draw ﬁrm conclusions from the limited sample size. In-depth metabolic characterization of bioﬂuids from patients with a range of pancreatobiliary conditions is required to further characterize metabonomic signatures for CCA.
